# Morphological Evolution of Sn‐Metal‐Based Anodes for Lithium‐Ion Batteries Using Operando X‐Ray Imaging

**DOI:** 10.1002/advs.202414892

**Published:** 2025-01-17

**Authors:** Bouchra Bouabadi, André Hilger, Paul H. Kamm, Tillmann R. Neu, Nikolay Kardjilov, Michael Sintschuk, Henning Markötter, Thomas Schedel‐Niedrig, Daniel Abou‐Ras, Francisco García‐Moreno, Sebastian Risse

**Affiliations:** ^1^ Department of Microstructure and Residual Stress Analysis Helmholtz‐Zentrum Berlin für Materialien und Energie Hahn‐Meitner‐Platz 1 14109 Berlin Germany; ^2^ Institute for Electrochemical Energy Storage (CE‐IEES) Helmholtz‐Zentrum Berlin für Materialien und Energie Hahn‐Meitner‐Platz 1 14109 Berlin Germany; ^3^ Bundesanstalt für Materialforschung und ‐Prüfung 12205 Berlin Germany; ^4^ Department of Structure and Dynamics of Energy Materials (SE‐ASD) Helmholtz‐Zentrum Berlin für Materialien und Energie Hahn‐Meitner‐Platz 1 14109 Berlin Germany

**Keywords:** foam electrodes, metal foil electrodes, operando X‐ray imaging, Sn‐based anodes

## Abstract

Sn‐based electrodes are promising candidates for next‐generation lithium‐ion batteries. However, it suffers from deleterious micro‐structural deformation as it undergoes drastic volume changes upon lithium insertion and extraction. Progress in designing these materials is limited to complex structures. There is a significant need to develop an alloy‐based anode that can be industrially manufactured and offers high reversible capacity. This necessitates a profound understanding of the interplay between structural changes and electrochemical performance. Here, operando X‐ray imaging is used to correlate the morphological evolution to electrochemical performance in foil and foam systems. The 3D Sn‐foam‐like structure electrode is fabricated in‐house as a practical approach to accommodate the volume expansion and alleviate the mechanical stress experienced upon alloying/dealloying. Results show that generating pores in Sn electrodes can help manage the volume expansion and mitigate the severe mechanical stress in thick electrodes during alloying/dealloying processes. The foam electrode demonstrates superior electrochemical performance compared to non‐porous Sn foil with an equivalent absolute capacity. This work advances the understanding of the real‐time morphological evolution of Sn bulky electrodes.

## Graphical abstract

A 3D Sn‐foam‐like structure electrode, designed as an anode for Li‐ion batteries, is fabricated in‐house as a practical strategy to mitigate the mechanical stress associated with alloying/dealloying processes in Sn‐based anodes. Employing operando X‐ray imaging, a correlation is established between the morphological transformations and the electrochemical performance of Sn foil and foam systems, facilitating a comparative analysis.

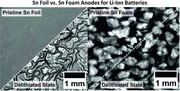

## 1 Introduction

Sn is a potential candidate for high energy density Li‐ion batteries (LIBs) with a theoretical gravimetric capacity of 993 mAh g^−1^ compared with conventional graphite 372 mAh g^−1^.^[^ ^1^, ^2^ ^]^ It is considered among the alloying anode materials (Si, Ge, Sb) that have the highest likelihood of being implemented in commercial cells.^[^ ^3^, ^4^ ^]^ Although Sn forms alloys with Li as Si and Ge, the ductility and malleability properties of Sn make its lithiation and degradation mechanisms different compared to these brittle semiconductors.^[^ ^5^ ^]^ Sn material has attracted significant attention owing to its high specific capacity, low potential versus Li metal, electrical conductivity, low cost, and abundance.^[^ ^1^, ^4^, ^6^ ^]^ However, the colossal morphological and structural changes associated with the volume expansion/contraction during its lithiation/delithiation can cause substantial irreversible damage to the electrode matrix, hindering its application. The alloy‐based material experiences considerable Li diffusion‐induced stress (LDIS) during Li insertion/extraction, generating cracks and fractures.^[^ ^5^, ^6^, ^7^, ^8^ ^]^ The repeated exposure of the fractured active material to electrolyte causes continuous growth of solid electrolyte interphase (SEI) layers, resulting in their electrical disconnection and subsequent crumbling into inactive powder, leading to capacity loss.^[^ ^1^, ^5^, ^6^, ^7^, ^9^, ^10^, ^11^ ^]^ Thus, considerable effort over the past decade has been dedicated to understanding and developing new strategies for bypassing the aforementioned issues.

Nanoscaling and/or fabricating porous structures are among the most studied approaches to prevent cracking and buffer the volume expansion in these materials.^[^ ^6^, ^12^, ^13^, ^14^, ^15^, ^16^ ^]^ Porous nano‐structured electrodes showed promising results as the void in these structures can accommodate the volume expansion and relieve the mechanical stress, enhancing the cycle performance.^[^ ^17^, ^18^, ^19^, ^20^, ^21^ ^]^ However, the intricate fabrication methods of these engineered porous nanostructures hinder large‐scale manufacturing and impede the advancement of alloy‐based anodes.^[^ ^7^ ^]^ Therefore, an anode based on an alloy material that can be manufactured on an industrial scale with reversible capacity needs to be designed. Studies have attempted to develop macroscale dense films and foil‐based anodes as they exhibit a high volumetric capacity and are easy to fabricate on a large scale.^[^ ^22^, ^23^, ^24^, ^25^, ^26^, ^27^, ^28^ ^]^ However, it is still a daunting task, as these thick materials can undergo considerable mechanical stress during their lithiation, causing severe structural changes that ultimately lead to their pulverization. The interplay between the electrochemical performance and morphological change of these dense Sn electrodes during the lithiation/delithiation is still not well understood. A deep understanding of the spatio‐temporal LDIS evolution and its correlation with electrochemical potential in various bulky electrode geometries is essential to engineering practical dense Sn‐based electrodes. Operando imaging is an excellent tool that can help to elucidate how the morphological and structural transformations evolve during lithiation/delithiation in real time.^[^ ^29^, ^30^, ^31^ ^]^

In the present work, we used operando X‐ray imaging to observe the interplay between the morphological evolution and electrochemical performance of Sn disk‐like foils with a 14 mm diameter (25 and 100 µm thickness). Sn foils were cycled at a current of 1.54 mA (1 mA cm^−2^), applying the same areal current density under the assumption that the lithiation occurs predominantly at the surface, as both electrodes were partially lithiated. An Sn foam‐like architecture electrode was fabricated to circumvent the huge volume expansion and strain generated in Sn foil electrodes due to Li insertion. The morphological and electrochemical performance of foil and foam electrodes with similar capacities were compared. The foam electrode, with ≈50% porosity, 200 µm in thickness, and 14 mm in diameter, exhibited better performance and maintained its overall structure over many cycles, compared to the foil electrode (100 µm in thickness and 14 mm in diameter), which experienced severe structural degradation after only a few cycles. Since both electrodes have similar absolute capacities, the same gravimetric current density was applied. The porosity of the foam electrode not only accommodates the expansion volume during lithiation but also facilitates higher electrolyte content. It also shortens the Li diffusion and electron path lengths. All the electrodes are devoid of any binder or additives. Fluoroethylene carbonate (FEC) was not added to the electrolyte, despite its known benefits of enhancing capacity retention and reducing electrochemical impedance on Sn electrodes,^[^ ^11^, ^32^ ^]^ to exclude its effect on the electrode performance. The capacity of the electrodes was deliberately restricted to minimize the violent changes the electrodes undergo, thereby allowing the observation of gradual structural changes and finer details that can be obscured during cycling. The X‐ray images show the expansion, contraction, and cracking behaviors of the Sn foil and foam electrodes during lithiation/delithiation processes. Our results demonstrate that the porous structure of bulky electrodes enables the accommodation of the volume expansion and consequently lessens the morphological changes. The grain size of the foam electrodes is much smaller than that of the foil, which is believed to contribute to its better performance. However, accommodation of the volume expansion is not enough to avoid cell failure. Modulating the foam wall thickness to avoid cracks, along with SEI engineering to prevent its rupture and reformation, is still needed. This work provides important insights into morphological evolution and electrochemical potential interrelation, which is difficult to obtain with ex‐situ methods. The insights gained here can help to model the LDIS evolution and cracking behavior of these materials and to design crack‐free LIB electrodes.

## 2 Results and Discussion

### 2.1 Morphological Evolution of Sn Foil Electrodes During Lithiation/Delithiation

**Figure 1:**
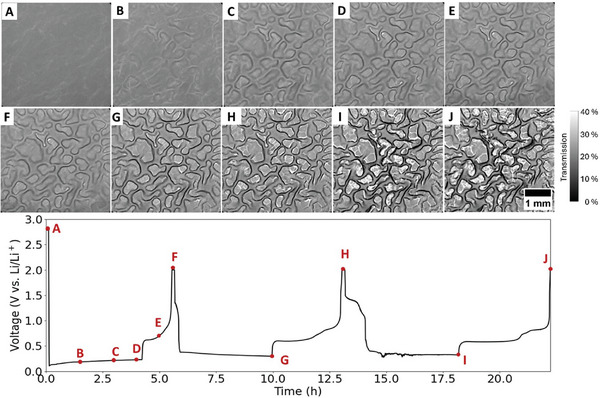
2D morphological evolution and galvanostatic discharge/charge profile of Sn foil electrode (14 mm diameter and 25 µm thick) cycled in a half‐cell configuration against Li counter‐electrode during the first three cycles. The cell was discharged/charged at a current of 1.54 mA (54.7 mA g^−1^), with the lithiation process limited to four hours. Frames (A–J): Lab‐based X‐ray images captured at different stages of lithiation/delithiation process. The specific time points at which these images were acquired are indicated on the corresponding voltage profiles.

**Figure 2:**
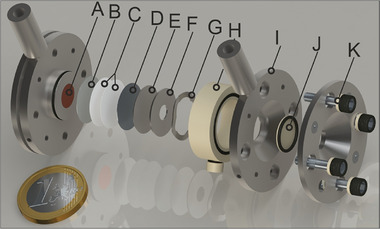
Schematic of the operando cell architecture for the discharge/charge experiment. A) Current collector, B) Sn electrode, C) separator (Celgard), D) lithium chip, E) steel spacer, F) steel spacer, G) steel spring, H) polyether ether ketone (PEEK) ring, I) stainless steel electrode, J) PEEK window, and K) stainless steel screw.

**Figure** **1** displays the voltage profile of the first three cycles of a disk‐shaped Sn foil (14 mm in diameter and 25 µm in thickness) during lithiation/delithiation, along with a series of laboratory‐X‐ray images selected at different stages of these cycles. The electrode was cycled in a half cell against a Li metal using an electrolyte of 1 m LiPF_6_ in a 50:50 volume ratio solution of ethylene carbonate (EC) and dimethyl carbonate (DMC). The cell was assembled according to the arrangement shown in the schematic in **Figure** **2**. The electrode was lithiated and delithiated using a galvanostatic process, with a constant current of 1.54 mA (1 mA cm^−2^, 54.7 mA g^−1^) applied for four hours during lithiation. The cycling rate was ≈ C/18, with a maximum charge voltage set at 2 V. The capacity was restricted to 218.9 mAh g^−1^, which corresponds to 22% of its theoretical capacity. Assuming that the lithiation process of Sn initiates at ≈0.8 V, and above this voltage, the surface of Sn merely catalyzes the decomposition of the electrolyte,^[^ ^2^, ^33^ ^]^ the duration of the lithiation process for the electrodes was measured starting from 0.8 V. The initial Coulombic efficiency (ICE) is 33%. Such considerable initial irreversible loss (67%) that occurs on Sn‐based anode materials is typically ascribed to the decomposition of the electrolyte, formation of solid electrolyte interphase (SEI) layer on the anode surface, and the incomplete dealloying of the anode as the lithium gets trapped within the electrode.^[^ ^6^, ^15^, ^30^, ^33^, ^34^ ^]^ The SEI layer consists mainly of Li_2_CO_3_ and lithium alkyl carbonates (ROCO_2_Li).^[^ ^34^, ^35^, ^36^ ^]^ Owing to the presence of the native oxide layer on the Sn surface due to its exposure to air, Li initially reacts with the oxidized layer to form Li_2_O, contributing to the irreversible Coulombic efficiency (CE) and subsequently lithiates with the bulk Sn.^[^ ^2^, ^3^, ^37^, ^38^ ^]^ The CEs of the second and third cycles are 79.25% and 99%, respectively. The noise observed in the discharge curve during the third cycle is most likely attributed to the transient partial loss of electrical contact at the Sn/current collector interface, caused by the substantial morphological changes occurring during the lithiation process. The gradual enhancement of the CE observed over the three charge/discharge cycles is believed to be attributed to the reversibility of the Li‐Sn compounds formed in the preceding cycle, as well as Li reaching fresh new areas. Conversely, the accumulated capacity loss during cycling is hypothesized to stem from the continuous formation of SEI layer, due to the recurrent exposure of fresh areas to the electrolyte, as well as the emergence of new irreversible phases at each cycle. Figure S1 (Supporting Information) displays the galvanostatic discharge/charge profiles and the corresponding capacities of a 25 µm thick foil cycled off‐beam. The inset values in the capacity plot (middle row) represent the Coulombic efficiencies of their respective cycles. Figure S2 (Supporting Information) presents images of the foil electrode before and after 20 cycles (sample cycled in Figure S1, Supporting Information).

In the initial lithiation, Figure 1, the voltage rapidly drops to low values of ≈0.11 V versus Li^+^/Li. This drop can be associated with the nucleation of a new phase.^[^ ^39^ ^]^ The voltage then increases with a plateau‐like curve, reaching a voltage of 0.23 V versus Li^+^/Li after four hours of lithiation. The plateauing voltage is a signature of metallurgical phase transformation and the coexisting of two phases during alloying/dealloying, whereas the sloping of the voltage curve indicates single‐phase formation.^[^ ^40^, ^41^, ^42^, ^43^ ^]^ During the subsequent cycles, the lithiation voltages shifted to higher voltages, which is believed to be due to stress relaxation. The relatively flat voltages during our cycling suggest the involvement of a two‐phase mechanism throughout most of the lithiation processes. On the other hand, the first distinct voltage step emerged during the initial delithiation, and a second faint voltage step began to form in the subsequent delithiations. With continued cycling, both steps became more pronounced (Figure S1 Supporting Information). This is further supported by electrochemical impedance spectroscopy (EIS) measurements, which show a clear distinction between the first lithiated state and the ones in the subsequent two cycles (Figures S3–S5, Supporting Information). Based on the thermodynamic properties of the Li‐Sn system, obtained from experimental thermodynamics data using Gibbs free energy,^[^ ^44^ ^]^ and the galvanostatic voltage profile, one should be able to predict the phase formed at different stages of lithiation. However, the recorded potentials are lower than the thermodynamically expected values. This discrepancy is explained by the contribution of elastic‐strain energy to the chemical free energy of the formation of different Li*_x_* Sn phases.^[^ ^40^, ^45^, ^46^, ^47^ ^]^ Ichitsubo et al.^[^ ^46^, ^47^ ^]^ estimated the elastic‐strain energy contribution using micromechanics theory based on Eshelby's ellipsoidal inclusion. It demonstrates that the electrode potential corresponding to a specific Li*_x_* Sn phase tends to be lower than the value predicted by considering solely Gibbs free energy.^[^ ^46^, ^48^ ^]^ It is worth noting that the voltage drop due to the elastic‐stress accommodation can be on the order of 0.5 V.^[^ ^46^, ^49^ ^]^ It also suggests that the formation of Li*_x_* Sn phases with higher Li concentrations on the surface is thermodynamically more favorable than the formation of Li*_x_* Sn phases with low Li content within the electrode.^[^ ^46^ ^]^ The elastic strain energy associated with the formation of Li‐Sn compounds at the surface is easily released compared to the ones in the interior.^[^ ^46^ ^]^ Therefore, the observed low voltages during lithiation in the three cycles can be explained by both the contribution of the elastic‐strain energy and the formation of Li‐rich Li*_x_* Sn phases on the surface. It is worth mentioning that reaction kinetics is influenced by the lithiation rate, the cell arrangement, and therefore affecting the Li*_x_* Sn phase formed.

To understand how LDIS evolves in Sn‐based dense anodes during lithiation/delithiation, operando laboratory X‐ray imaging was carried out. This non‐destructive method enables real‐time observation of the morphological variations in the Sn anode. The images in Figure 1 show the morphological changes of the 25 µm thick Sn foil electrode during the first three cycles at different stages of its lithiation/delithiation. The times when images were taken are shown on the voltage profile. After one hour of lithiation at a current of 54.7 mA g^−1^, Sn foil experiences massive mechanical stress leading to plastic deformation exhibiting a faint multi‐zone pattern (Figure 1B). Sn, like other metals, can accommodate a certain level of mechanical stress induced during lithiation. The LDIS in alloy material arises primarily due to changes in lattice dimensions, modification in crystal structure, and phase transformation.^[^ ^43^, ^49^ ^]^ When the extensive volume changes during lithiation surpass the elastic limit, the material undergoes plastic deformation. The produced pattern consists of non‐uniform fragments, called here islands, peripheries surrounding these islands, and interconnected spaces called floors. As the amount of lithium inserted increases during the initial lithiation, the faint pattern becomes more pronounced and the periphery darkens. Over the subsequent discharge/charge cycles, cracks and voids appear within the islands, the floor shrinks, and the periphery thickens (Figure 1B–J; see also Video S1, Supporting Information). The behavior within the islands is consistent with the lithiation/delithiation behavior of Sn particles reported in the literature,^[^ ^6^, ^15^, ^30^ ^]^ suggesting that most alloying/dealloying process occurs within the islands. In contrast to ductile Sn metal, Li*_x_* Sn is brittle,^[^ ^15^, ^50^, ^51^ ^]^ making it more prone to cracking under sufficient stress.^[^ ^15^ ^]^ The floor regions exhibit almost no observable cracks or void development over the three cycles, suggesting that these areas are either not lithiated or contain Li*_x_* Sn phases with low lithium content. The formation of Li*_x_* Sn phases is indicated by a decrease of X‐ray attenuation coefficient during the lithiation of the Sn electrode.^[^ ^31^ ^]^ The calculated average transmission coefficients and phase fractions across the three distinct regions in the electrode are presented in Figure S6 (Supporting Information). Within the islands, the transmission coefficient shows an increase over cycling, which is attributed to the change in composition and the crack growth. Conversely, the floor area shows an increase in the X‐ray transmission during the initial lithiation, indicating a change in chemical composition from Sn to Li*_x_* Sn, and then remains relatively steady throughout the subsequent cycles. The transmission through the peripheries decreases over cycling, suggesting high mechanical stress and probably surface wrinkling. Accordingly, it is believed that the concentrated mechanical stress in the floor and peripheries fails to be relieved, as the formation and release of stress in lithiated areas in Sn materials lead to void creation.^[^ ^31^ ^]^ Owing to the formation of cracks and voids in the islands, the electrolyte has easy access to the interior, thereby accelerating the lithiation process (see Video S1, Supporting Information). This is also confirmed by the decreasing charge transfer resistance in the lithiated state of the subsequent cycles, as measured by EIS shown in Figures S3–S5 (Supporting Information). The data was fitted with a distribution of relaxation times model (Supporting Information). However, as the islands disintegrate into small pieces due to fracture, the electron supply to these pieces is interrupted, leading to their inactivity. Conversely, the lack of electrolyte ingress to the floor areas due to the unrelieved mechanical stress affects the lithiation kinetics, impeding any further Li diffusion.

**Figure 3:**
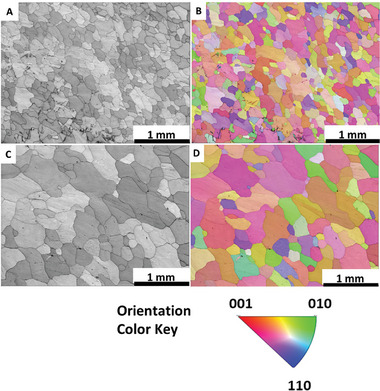
Scanning electron microscopy (SEM) images and electron backscattered diffraction (EBSD) orientation‐distribution maps (the local orientations are given as false colors, see legends) images. Sn foil electrode (14 mm in diameter and 25 µm in thickness) A) SEM B) EBSD. Sn foil electrode (14 mm in diameter and 100 µm in thickness) C) SEM D) EBSD.

Mathematical models have been developed to elucidate the evolution of LIDS and the resultant cracking phenomena in materials such as Si‐thin films, which exhibit characteristic mud‐cracking patterns.^[^ ^8^, ^52^ ^]^ However, the ductility and malleability properties of Sn result in lithiation and degradation mechanisms that differ from those brittle semiconductors,^[^ ^5^ ^]^ displaying an intriguing pattern. The modeling of pattern formation, crack evolution, and the shrinkage process have yet to be established for this ductile material. Here, we limited the capacity to ensure the electrode undergoes less extensive and potentially less violent changes, which can often obscure the finer details responsible for its degradation. When these changes are moderated, it becomes easier to pinpoint and observe specific morphological features and alterations that evolve under less severe, yet still relevant, conditions. It also reveals how much the system can withstand during lithiation/delithiation across various stages of stress, particularly with cumulative capacities, before breaking down. The obtained pattern revealed by X‐ray imaging (Figure 1J; see also Video S1, Supporting Information) suggests the presence of preferential areas where Li initially penetrates, and the subsequent lithiation occurs based on these initial choices, resulting in an inhomogeneous structure with uneven Li concentrations. Therefore, the pattern is believed to depend on the initial texture of the Sn foil, with Li infiltrating certain grains along specific planes faster than others. The resulting pattern compelled us to investigate the initial texture of our electrode. **Figure** **3**A,B show the scanning electron microscopy (SEM) and the electron backscattered diffraction (EBSD) images of the grain distribution in pristine Sn foil electrode, 25 µm thick, where grains with an average grain size of 115 µm are visible. Initially, the Li diffusion is supposed to occur primarily along the grain boundaries (GBs).^[^ ^23^, ^53^, ^54^, ^55^, ^56^ ^]^ The GBs act as conductive binders,^[^ ^57^, ^58^ ^]^ connecting the grains and enabling their electrochemical conductivity. However, a small liquid electrolyte quantity can infiltrate GBs, forming a SEI layer. If this SEI layer becomes sufficiently thick in the GBs, it will impede further electronic conductivity. In our 25 µm thick foil, the average grain size is larger than its thickness, which may allow Li to go along GBs to the bottom in the initial lithiation. However, the generated islands are larger than the individual grains, suggesting the islands consist of clusters of grains. It is believed that although the initial percolation of Li may proceed along the GBs, subsequent lithiation/delithiation occurs mainly in the preferred grains or cluster of grains. The islands retained their overall original shape, which can be attributed to the fact that Li diffuses faster along preferred directions,^[^ ^59^ ^]^ profoundly influencing the ultimate pattern. The GBs in the floor area are expected to undergo sliding and dislocations for stress relaxation, which are the primary mechanisms for absorbing large mechanical stresses.^[^ ^23^, ^25^ ^]^ Figure 3D shows the EBSD of Sn foil with 14 mm in diameter and 100 µm in thickness. The foil is composed of well‐defined grains with an average size of 260 µm. As expected, a larger grain size (260 vs 115 µm) was found for the thicker Sn foil (100 vs 25 µm). No significant differences were observed between the two Sn foils in terms of the local grain orientations. Video S2 (Supporting Information) suggests that the lithiation in the 100 µm thick foil occurs primarily via grain boundaries.

As explored by Obrovac et al.^[^ ^60^, ^61^ ^]^ Li occupies approximately a molar volume of 9 mL mol^−1^ in a wide variety of lithium alloys, independently of the Li content and the host material. The insertion of Li in alloy materials follows Vegard´s law.^[^ ^60^ ^]^ The molar volume of Li*_x_* M was derived from the Equation (1):^[^ ^60^ ^]^

(1) $$V=xk+v_{0}$$

where *V* is the molar volume of the Li*_x_* M alloy per metal, *v_0_* is the molar volume of the pure metal M, and *k* is the molar volume of Li in the Li*_x_* M. The percent volume expansion follows a linear relationship with Li content. Sn can theoretically host up to 4.4 Li atoms per Sn atom, corresponding to the fully lithiated phase Li_4.4_Sn. Determining the exact value of *x* in the formed Li*_x_* Sn is difficult due to the consumption of Li to form the SEI layer and the non‐uniform distribution of Li concentration within the electrode, as suggested by the X‐ray images. The Sn foil electrode was lithiated to 22% of its theoretical capacity, corresponding to the Li*_x_* Sn phase with a maximum value of *x* = 0.97. The *x* value was estimated by assuming that all the Li inserted into the electrode forms Li_0.97_Sn phases and that the distribution is homogeneous. Using Equation (1), the calculated total molar volume expansion is 53.57% relative to pristine Sn. The unevenly distributed mechanical stress and lithiation (Figure 1J), resulting in localized volume expansions. This implies that there are regions with Li*_x_* Sn phases having higher Li content (*x* > 0.97), which was confirmed by low lithiation voltages, and others have lower content (*x* < 0.97).

**Figure 4:**
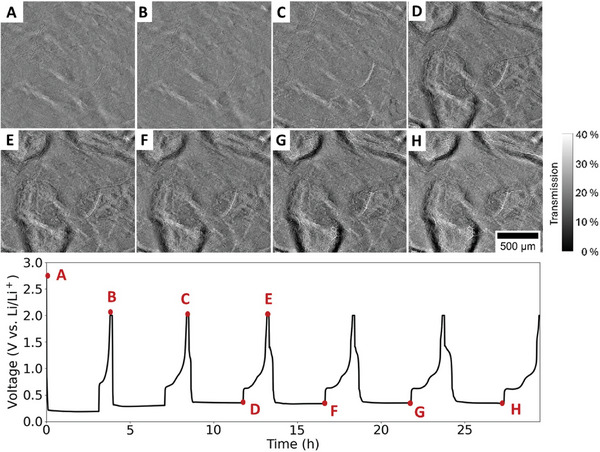
2D morphological evolution and galvanostatic discharge/charge profile of Sn foil electrode (14 mm diameter and 25 µm thickness) cycled in a half cell against Li counter‐electrode during the first six cycles. The cell was discharged/charged at current of 0.5 mA (17.77 mA g^−1^), with the lithiation process limited to four hours. Frames A–H): Synchrotron X‐ray images taken at various stages of lithiation/delithiation process. The specific time points at which these images were acquired are indicated on the corresponding voltage profiles.

To gain further information on the finer structures, operando synchrotron X‐ray imaging was performed. Its spatial resolution and contrast allow the observation of the subtle and fine changes in Sn foil material during lithiation/delithiation, which are beyond the detection capability of conventional laboratory‐based X‐ray sources. An Sn foil electrode, 25 µm in thickness and 14 mm in diameter was partially lithiated by passing a current of 0.5 mA (17.77 mA g^−1^) for three hours, restricting it to 5.37% of its theoretical capacity. **Figure** **4** displays the voltage profile of the first six cycles of the Sn foil electrode, 25 µm in thickness and 14 mm in diameter, during lithiation/delithiation, accompanied by a series of X‐ray images, selected at different stages of these cycles. The times when images were taken are shown on the voltage profile. The voltage of initial lithiation dips to ≈0.2 V and then plateaus at that potential for three hours. The lithiation rate was lower than the one above, resulting in higher lithiation voltage. It has been reported that in the Sn‐Li systems, the electrode potential depends on the current density, decreasing as the current increases,^[^ ^45^ ^]^ which was confirmed here. The CE values for the six consecutive cycles are 33.33%, 50%, 52%, 59%, 66.67%, and 70%, respectively, with a total accumulated capacity over the six cycles of 4.04 mAh (143.57 mAh g^−1^). The electrode potential for each lithiation cycle rises as the number of cycles increases. The degree of the initial lithiation is approximately *x* = 0.24, with a total percent volume expansion of ≈13.04%. Upon the completion of the first cycle, no noticeable morphological change was observed (Figure 4A), implying that the electrode accommodates the elastic stress produced by a charge insertion of ≈5.4% of its capacity. The electrode could bear this stress level and release it without any visible structural change, with an irreversible capacity of 1 mAh (35.54 mAh g^−1^). At the end of the second lithiation, the total charge stored in the electrode is 2.5 mAh (88.84 mAh g^−1^), which is the same amount of charge stored in the electrode lithiated at a higher rate, where the electrode exhibits plastic deformation (Figure 1B). However, the electrode still did not show morphological change, which could be ascribed to the lower current density, allowing the electrode to accommodate and release stress without apparent plastic deformation. During the second lithiation patches on the surface of the foil begin to appear. At the beginning of the third lithiation, liquid starts to flow over the surface (see Video S3, Supporting Information). Further insertion of Li into the electrode causes the islands to form, pushing the electrolyte to the floor, and forming the island's peripheries (Figure 4D; see also Video S3, Supporting Information). The morphological changes initiate during the third lithiation. Figure 4D displays the formed pattern, consisting of three distinguishable zones akin to the foil electrode above (Figure 1B), with a total charge insertion of 3.25 mAh (115.5 mAh g^−1^) by the end of the third lithiation. Cracks are observed within the islands during the third delithiation. During each subsequent delithiation process, the primary cracks widen, and new ones are generated, whereas no cracks are apparent in the floor (Figure 4D–H; see also Video S3, supporting information). The peripheries darkened over cycling, while the floor remained almost crack‐free. The expansion of the grains or a cluster of grains is believed to occur vertically. It is worth mentioning that gassing behavior on the surface was detected during the fifth lithiation, which contributes to the Sn electrode degradation mechanism.^[^ ^2^ ^]^ Using a lower cycling rate, limited capacity, and high spatial resolution enabled the observation of lithiation/delithiation stages at which morphological changes occur during cycling. The higher resolution of the synchrotron allowed the capture of liquid wetting and gassing behavior. The results indicate that stress development was more pronounced in the Sn foil that underwent faster lithiation. However, the electrodes displayed similar pattern shapes, suggesting that the pattern generation is not spontaneous. A more detailed study is needed to understand the mechanical stress evolution that leads to the formed pattern in Sn foil‐based systems and to determine which grain orientation best facilitates Li diffusion.

### 2.2 Morphological Evolution of Sn Foam Electrodes During Lithiation/Delithiation

**Figure 5:**
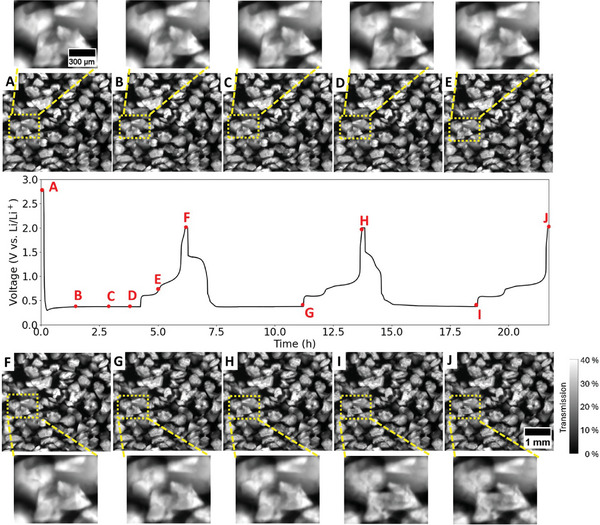
2D morphological evolution and galvanostatic discharge/charge curves of Sn foam electrode (14 mm diameter and 200 µm thickness) cycled in a half‐cell configuration against Li counter‐electrode during the first three cycles. The cell was discharged/charged at current of 1.54 mA (14.9 mA g^−1^), with the lithiation processes limited to four hours. Frames (A–J): Laboratory‐based X‐ray images captured at different stages of lithiation/delithiation process. The specific points at which these images were acquired are indicated on the corresponding voltage profiles.

**Figure 6:**
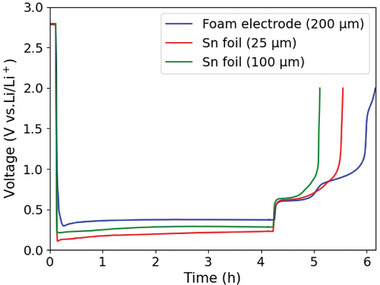
Galvanostatic discharge/charge curves of the first cycle for Sn foam (200 µm thickness) and Sn foils (25 and 100 µm thick) cycled in a half‐cell configuration against Li counter‐electrode. The cell was discharged/charged at a current of 1.54 mA, with the lithiation process limited to four hours.

**Figure 7:**
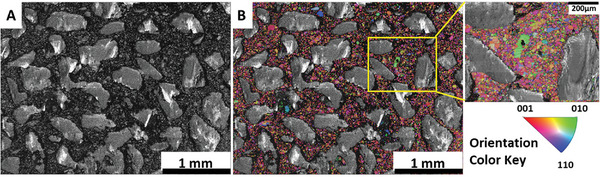
Scanning electron microscopy (SEM) image and electron backscattered diffraction (EBSD) orientation distribution map acquired on a Sn foam electrode B), the local orientations are given as false colors, see legends. This EBSD map is superimposed on an EBSD band‐contrast map.

In order to mitigate the mechanical stress experienced by foil electrodes, an Sn foam electrode was fabricated with 54% porosity to accommodate the volume expansion during lithiation. This electrode was compared with a non‐porous 100 µm thick foil, which has nearly the same absolute capacity. The pores are not only supposed to accommodate the volume expansion and help reduce the stress occurring during the lithiation, but they also provide a very high surface area, enhancing the electrolyte diffusion and shortening the paths of Li ions and electrons in the electrode to the thickness of the walls. The effect of the larger surface of the foam electrode is also reflected by the significantly lower charge transfer resistance measured with EIS at the end of the discharge and charge steps for the first three cycles (Figures S3–S5, Supporting Information). **Figure** **5** shows the voltage profile of the first three cycles of the Sn foam electrode, 14 mm in diameter and 200 µm in thickness, during lithiation/delithiation, accompanied by a series of X‐ray images selected at different stages of these cycles. The assembled half‐cell was cycled by performing a galvanostatic charge/discharge process at a current density of 1.54 mA (14.9 mA g^−1^). The lithiation process was limited to four hours, and the maximum charge voltage was set at 2 V. The capacity was restricted to 6%. The potential first dropped to 0.3 V, which is higher than the initial lithiation of the 25 µm and 100 µm foil lithiated at the same current (**Figure** **6**). The difference in the initial potential drop can be attributed to the significantly larger surface area of the foam electrode compared with foil‐based systems and the reduced contribution of the elastic‐strain energy. Figure S7 (Supporting Information) shows an SEM image of the Sn foam. The CE values for the three consecutive cycles of the foam electrode (Figure 5) are 48.25%, 62.75%, and 75.5%, respectively, resulting in a total accumulated capacity of 4.54 mAh (43.78 mAh g^−1^). The total volume expansion during the initial lithiation based on the inserted charge starting from 0.8 V, is estimated to be 14.59%. Consequently, there is sufficient space to absorb this volume expansion. The images (Figure 5A–J) demonstrate that the overall shape of the foam Sn‐electrode remains almost unchanged over three cycles, with subtle changes observed in the zoomed‐in images. Video S4 (Supporting Information) shows that after five cycles, there is still no significant change in the overall structure of the Sn foam‐like structure electrode. This observation is consistent with our prediction that the pores accommodate the volume expansion and buffer the mechanical stress. However, the foam electrode exhibits a breathing behavior during the expansion/contraction of Sn foam, with Sn expanding into the void space (see Video S4, Supporting Information). The expansion of Sn during lithiation does not occur gradually, instead, it progresses in distinct sub‐steps (see Figure S8, Supporting Information for more details). The lithiation in the foam electrode occurs mainly through the infiltration of the electrolyte via the pores, followed by the diffusion of Li‐ions from the surface of the walls to the core. In addition to the initial pores, the formed cracks in the Sn foam walls allow the permeation of electrolytes, accelerating the lithiation rate. Figure S9 (Supporting Information) illustrates the galvanostatic discharge/charge cycles of Sn foam electrode and their corresponding capacities over 25 cycles. The inset values in the capacity plot (middle row) represent the Coulombic efficiencies of their respective cycles. The voltage profiles show one plateauing voltage behavior during the lithiation processes, while two voltage steps are observed during the delithiation. While porosity plays a role in stress accommodation, it is also believed that the smaller grain size of the Sn foam walls, compared with that of Sn foils (Figure 3B,D), contributes to this effect. **Figure** **7**A,B displays SEM image and the grain distribution, showing tightly packed grains with an initial average size of 22 µm. The abundance of the GBs can help relieve stress due to their slippage and dislocations.^[^ ^23^ ^]^ The Li diffusivity is also expected to increase due to a higher abundance of GBs. Figure S10 (Supporting Information) presents images of the foam electrode before and after 25 cycles (sample cycled in Figure S9, Supporting Information).

The Sn foil electrode, 14 mm in diameter and 100 µm in thickness, with an absolute capacity comparable to that of the Sn‐foam electrode, was cycled at the same current of 1.54 mA, with the lithiation processes limited to four hours. Figure S11 (Supporting Information) displays the voltage profiles of the first three cycles of the Sn foil (14 mm in diameter and 100 µm in thickness) during lithiation/delithiation, along with a series of laboratory‐X‐ray images selected at different stages of these cycles (see also Video S5, Supporting Information). Videos S5 and S6 (Supporting Information) illustrate the undulation behavior of the 100 µm thick foil during volume changes, resulting in considerable damage to the electrode. The electrode has an initial grain size of 260 µm (Figure 3C), which explains the substantial mechanical stress it undergoes compared with the 25 µm foil (Figure 3B) and foam (Figure 6B) electrodes, which have relatively smaller grain sizes. Figure S12 (Supporting Information) displays the galvanostatic discharge/charge profiles and the corresponding capacities of a 100 µm thick foil cycled off‐beam. The inset values in the capacity plot (middle row) represent the Coulombic efficiencies of their respective cycles.

Figure 6 shows the initial lithiation and delithiation of the three systems: the Sn foam electrode (200 µm thick) and Sn foils (25 and 100 µm thick), which were lithiated at a constant current of 1.54 mA. The lithiation time was restricted to four hours, starting from a voltage of 0.8 V. Although the three systems were subjected to the same electrochemical protocol, the initial lithiation reaction occurred at different voltages. The reversible capacity is highly dependent on the initial structure and thickness of the Sn electrode, with the foam electrode demonstrating better reversibility, followed by the 25 µm foil. The plateauing voltage during the lithiation of the electrodes also exhibits sensitivity to the thickness and the architecture of the electrode. The superior cycling performance of Sn foam electrode, compared to the planar systems, results from the ability of its pores and smaller grain size to buffer the mechanical stress, enhancing the Li diffusivity during alloying/dealloying process.

Although the foam structure enables an even distribution of the electrolyte on the electrode surface and can alleviate the mechanical stress that Sn may experience, the cycle life remains limited by two primary factors: the formation of cracks in the foam walls and the instability of the formed (SEI) layer on the surface. When the thickness of the walls exceeds the critical size of Sn, they crack and eventually deteriorate. Furthermore, the SEI layer formed in the expanded state, fractures upon system contraction during dealloying. The repetition of this process leads to a thicker SEI layer and, ultimately, the inactivity of the material. Therefore, further optimization of the foam electrode is required to prevent the active material from cracking during the insertion and extraction of Li, by designing walls with thinner thickness and finer grains, maintaining the wall thickness below the critical size. Additionally, developing an SEI layer capable of withstanding repeated volume expansion and contraction is essential.

## 3 Conclusion

In the present study, real‐time morphological changes of foil and foam electrodes during lithiation and delithiation were monitored using operando X‐ray imaging techniques. The method has been shown to be useful in correlating mechanical behavior with the electrochemical performance of the alloy‐based electrodes. We attempted to address one of the critical challenges facing alloy anode materials: the volume expansion and drastic morphological changes caused by mechanical stress. The foam structure and its fabrication process represent an efficient approach for the development of advanced high‐energy density materials. Since metal foam production is already industrialized, this electrode exhibits high scalability potential. It was confirmed that the porous architecture and fine grains of Sn‐bulky systems mitigated mechanical stress on Sn‐based electrodes, increased the surface area, and facilitated electrolyte infiltration during lithiation, thereby improving the electrochemical performance of the anode. This study provides valuable insights into the structural changes of Sn foil during lithiation and delithiation, paving the way for the design of robust Sn‐based anodes for practical applications. Several experimental parameters must be optimized to develop a stable anode. Future investigations will focus on linking the initial grain distribution and size in Sn foil to the formed pattern and on how tuning these factors can enhance the electrochemical performance of the anode.

## 4 Experimental Section

### Materials Synthesis

An Sn foam‐like structure was fabricated for a scalable electrode using the powder metallurgical placeholder method.^[^ ^62^ ^]^ A mixture of Sn powder (Alfa Aesar, < 44 µm, purity 99.8%) and ammonium hydrogen carbonate (Alfa Aesar, sieved to 200–320 µm, purity 99%) was weighed in a ratio of 50:50 vol.% and mixed for 15 min in a drum hoop mixer. The powder mixture was then pressed at 300 MPa at room temperature to form a 20 mm diameter tablet. This was then sintered in two stages under vacuum for 2 h at 140 °C and 2 h at 180 °C: During this process, the ammonium hydrogen carbonate decomposed and left behind an open porous Sn sponge while the Sn particles sinter together and achieved mechanical stability.

### Electrochemical Measurements

The anode electrodes used in the present study were the in‐house manufactured Sn foam‐like structure and Sn foil electrodes. The Sn foil electrodes were purchased from Alfa Aesar (0.1 mm thickness, 99.99% purity) and Goodfellow (25 µm thickness, 99.95%); they were cut out with a punching die to obtain a disk‐shaped electrode of 14 mm diameter. 25 µm monolayer microporous membrane separators were purchased from Celgard. Li chips were purchased from MTI Corp with a thickness of 450 µm and a diameter of 15.4 mm. The Sn electrodes were cycled versus Li metal anode using an electrolyte of 1 m LiPF_6_ in a solution of ethylene carbonate (EC) and dimethyl carbonate (DMC) in a volume ratio of 50:50 purchased from Sigma Aldrich. The electrolyte was enough to wet the separator without flooding the cell. No binders or additives were used. The cells were assembled in an argon‐filled glovebox according to the arrangement shown in Figure 2. One stainless steel spring, two spacers, two layers of Celgard, and PEEK windows were used in the operando cell. The spring was used to ensure the optimal pressure in the cell. Spacers were used to maintain the flatness of the electrodes and prevent them from bending. The windows are made of PEEK to facilitate X‐ray transmission. A copper thin film of 200 nm was sputtered on the PEEK window on the Sn electrode side to enhance the current collection. All the Sn‐based electrodes have a disk shape with a diameter of 14 mm. The cells were subjected to a constant current during discharge/charge, with the lithiation process limited to four hours.

Potentiostatic electrochemical impedance spectroscopy was performed at the end of each charge and discharge step. The AC amplitude was set to 10 mV and the frequency ranges from 1 MHz to 100 mHz with 10 points per decade.

### Material Characterization

The structure and morphology of the foils and foam electrodes were characterized using X‐ray imaging. The laboratory X‐ray source imaging measurements were performed using a tungsten source. A beam energy of 80 kV and a current of 125 µA were set (no filter). The X‐ray images were acquired with an exposure time of 0.9 s on a panel detector (Hamamatsu, C7942K‐05, 23 162 316 active pixels) with a pixel size of 50 µm. The distance between the sample and the detector was 400 mm and between the source and the sample was 43 mm reflecting in magnification ratio of 9.3 x, which provides an effective pixel size of 5.4 µm. To minimize the X‐ray absorption PEEK windows were used.

Synchrotron X‐ray imaging experiments were conducted at BAM‐line, which was operated by the Federal Institute of Materials Research and ‐Testing, at the BESSY II electron storage ring, operated by Helmholtz‐Zentrum Berlin. Imaging was performed in monochromatic mode using a double multilayer monochromator with 70 bilayers of W/Si.^[^ ^63^ ^]^ The energy utilized was 35 keV with an energy resolution of ≈3–4%. The detector system comprises a 60 µm thick CdWO4 scintillator built in an Optique‐Peter White Beam Microscope and a PCO.Edge 5.5 sCMOS camera (2560 × 2160 pixel; PCO AG). Magnification was set to an effective pixel size of 0.72 µm. The distance between the detector and the sample was 40 cm. The individual frames were acquired with an exposure time of 2 s. In the analysis, several frames at the sample position were normalized with open beam images, which were taken in between. This sequence of sample radiographs and open beam images was repeated every 120 s, resulting in short interruptions in which the cell remains unobserved.

The analysis of the laboratory and synchrotron X‐ray images was performed using Fiji software.

The grain size and microstructure of Sn foam and foils were analyzed by means of electron backscatter diffraction (EBSD) using an Oxford Instruments Symmetry EBSD detector as well as the Aztec acquisition and evaluation software suite.

## Conflict of Interest

The authors declare no conflict of interest.

## Supporting information



Supporting Information

Supplemental Video 1

Supplemental Video 2

Supplemental Video 3

Supplemental Video 4

Supplemental Video 5

Supplemental Video 6

## Data Availability

The data that support the findings of this study are available from the corresponding author upon reasonable request.
