# Supplementary material for: Morphological Evolution of Sn‐Metal‐Based Anodes for Lithium‐Ion Batteries Using Operando X‐Ray Imaging
Source: Adv Sci (Weinh). 2025 Jan 17;12(10):2414892. doi: 10.1002/advs.202414892 (PMC11904996; doi:10.1002/advs.202414892)
Supplement: Supplementary file 1 — Supporting Information [file ADVS-12-2414892-s004.docx]

Supporting Information

**Morphological Evolution of Sn-metal-based Anodes for Lithium-Ion Batteries Using Operando X-ray Imaging**

*Bouchra Bouabadi*, André Hilger, Paul H. Kamm, Tillmann R. Neu, Nikolay Kardjilov, Michael Sintschuk, Henning Markötter, Thomas Schedel-Niedrig, Daniel Abou-Ras, Francisco García-Moreno, Sebastian Risse**


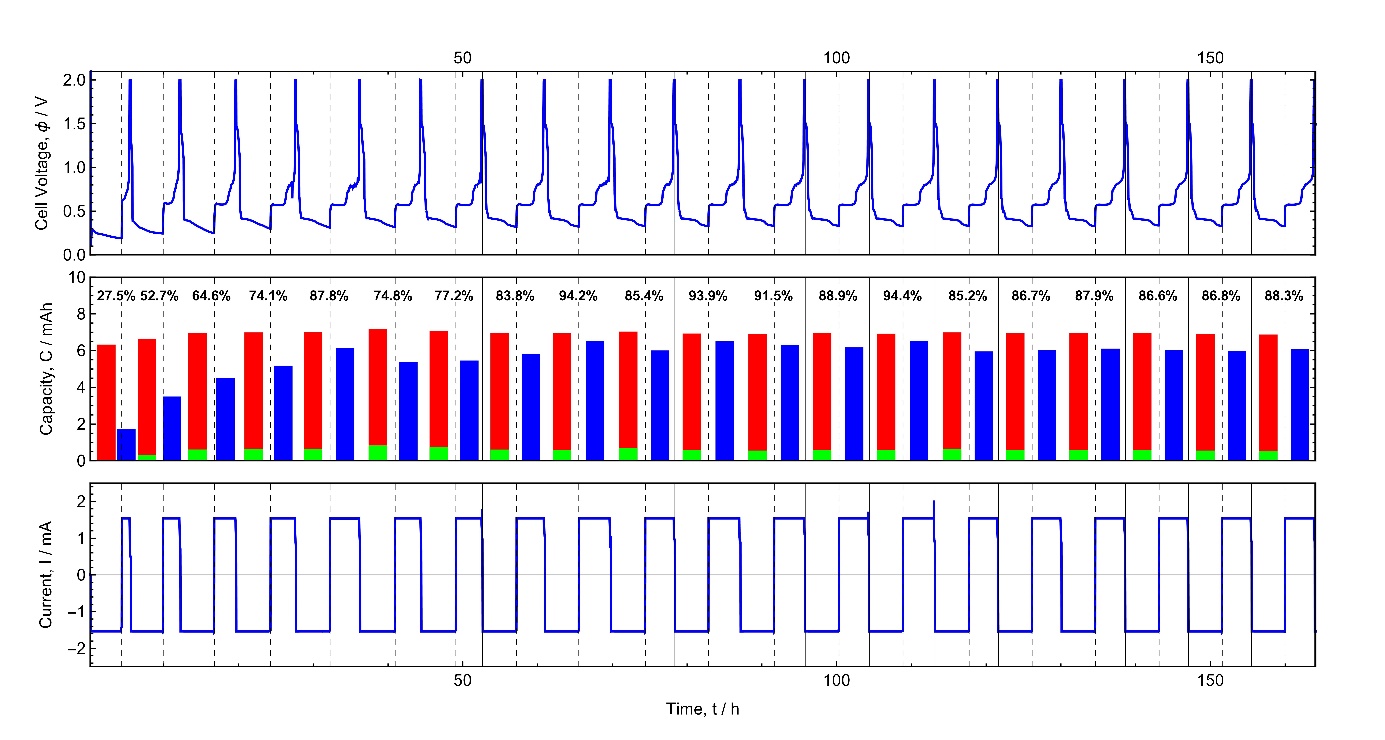
Figure S1: Galvanostatic discharge/charge profiles and the corresponding capacities of Sn foil electrode (25 µm thickness and 14 mm diameter) cycled in a half-cell configuration against lithium counter-electrode. The cell was discharged/charged at a current of 1.54 mA, with the lithiation process limited to four hours. Capacity: lithiation (green before 0.8 V, red starting from 0.8 V), delithiation (blue). The inset values in the capacity plot (middle row) represent the Coulombic efficiencies of their respective cycles.


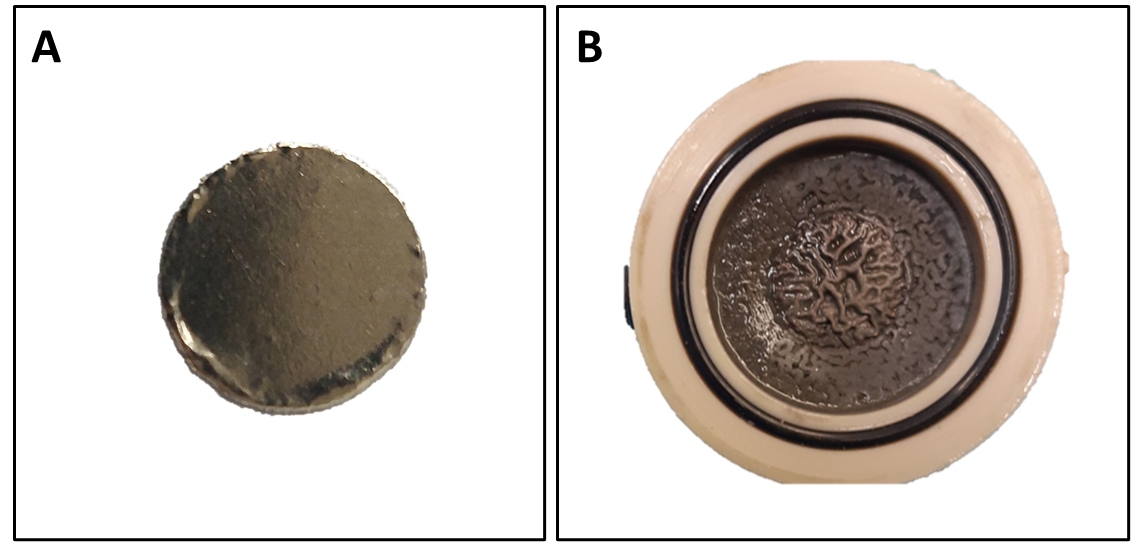


Figure S2: Images of Sn foil electrodes (25 µm thickness and 14 mm diameter): A) pristine and B) after cycling in a half-cell configuration against lithium counter-electrode. For B, the cell was discharged/charged at a current of 1.54 mA, with the lithiation process limited to four hours. This image corresponds to the sample shown in Figure S1 after 20 cycles.


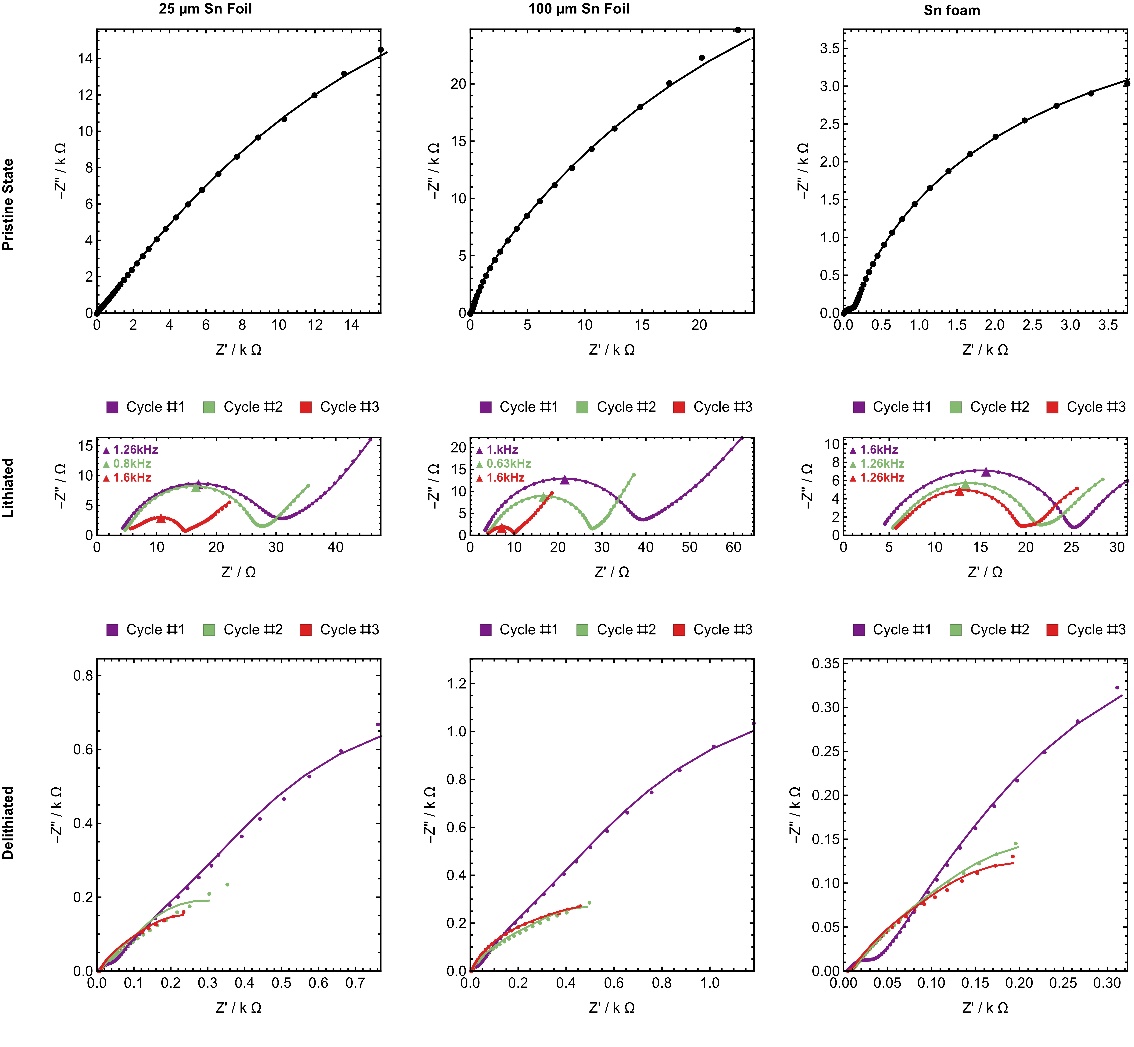


Figure S3: Overview of Nyquist plots of 25 µm Sn foil, 100 µm Sn foil, and Sn foam in the pristine state as well as the lithiated and delithiated states for the first three cycles. Triangles in the lithiated state plots show the relaxation frequencies of the observed charge transfer process. The solid lines represent the fits to the data using a distribution of relaxation times model.


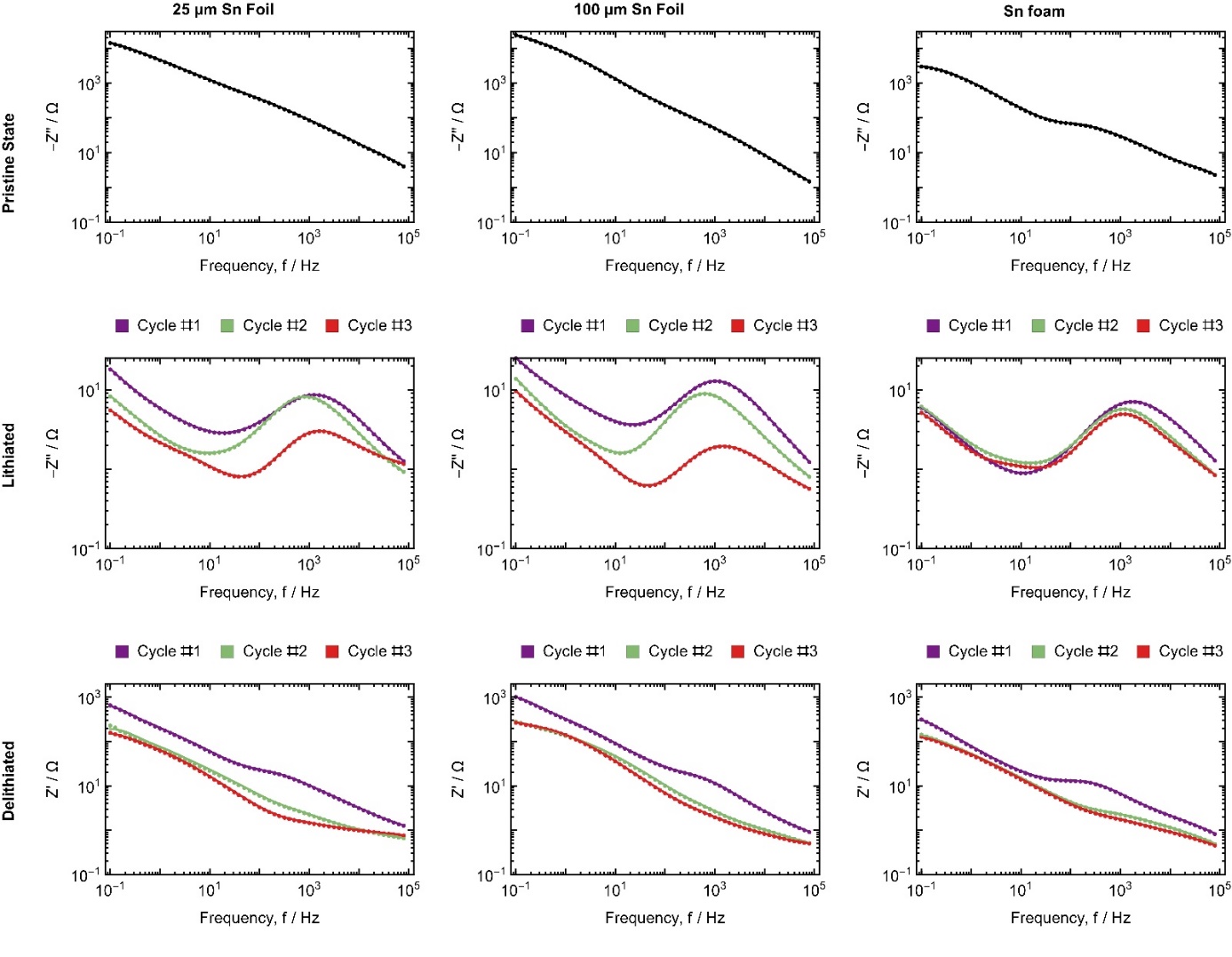


Figure S4: Overview of the spectra for the imaginary part of the impedance for 25 µm Sn foil, 100 µm Sn foil, and Sn foam in the pristine state as well as in the lithiated state and delithiated state for the first three cycles. The solid lines represent the fits to the data using a distribution of relaxation times model.

**Fitting the EIS to the distribution of relaxation times (DRT) model**

The EIS data in Figures S3 and S4 was fitted to the DRT model ($Z_{DRT}\left( \omega\right)$) by Wan et al ^[64].^ Gaussian distribution curves serve as radial basis functions. All data was fitted according to $Z_{DRT}\left( \omega\right)=R_{S}+\int_{-\infty}^{\infty} \frac{\gamma(\tau)}{1+i\omega\tau}d\ln\tau$ by using the regularization method described by Wan et al ^[64]^. We used $\lambda=1$ and $\mu={10}^{-6}$ as the regularization parameter and factor for the penalty term (M-matrix), respectively. The higher relaxation time ($\tau$) window was extended by 1.5 orders of magnitude to detect also relaxation processes slightly beyond the frequency observation interval.


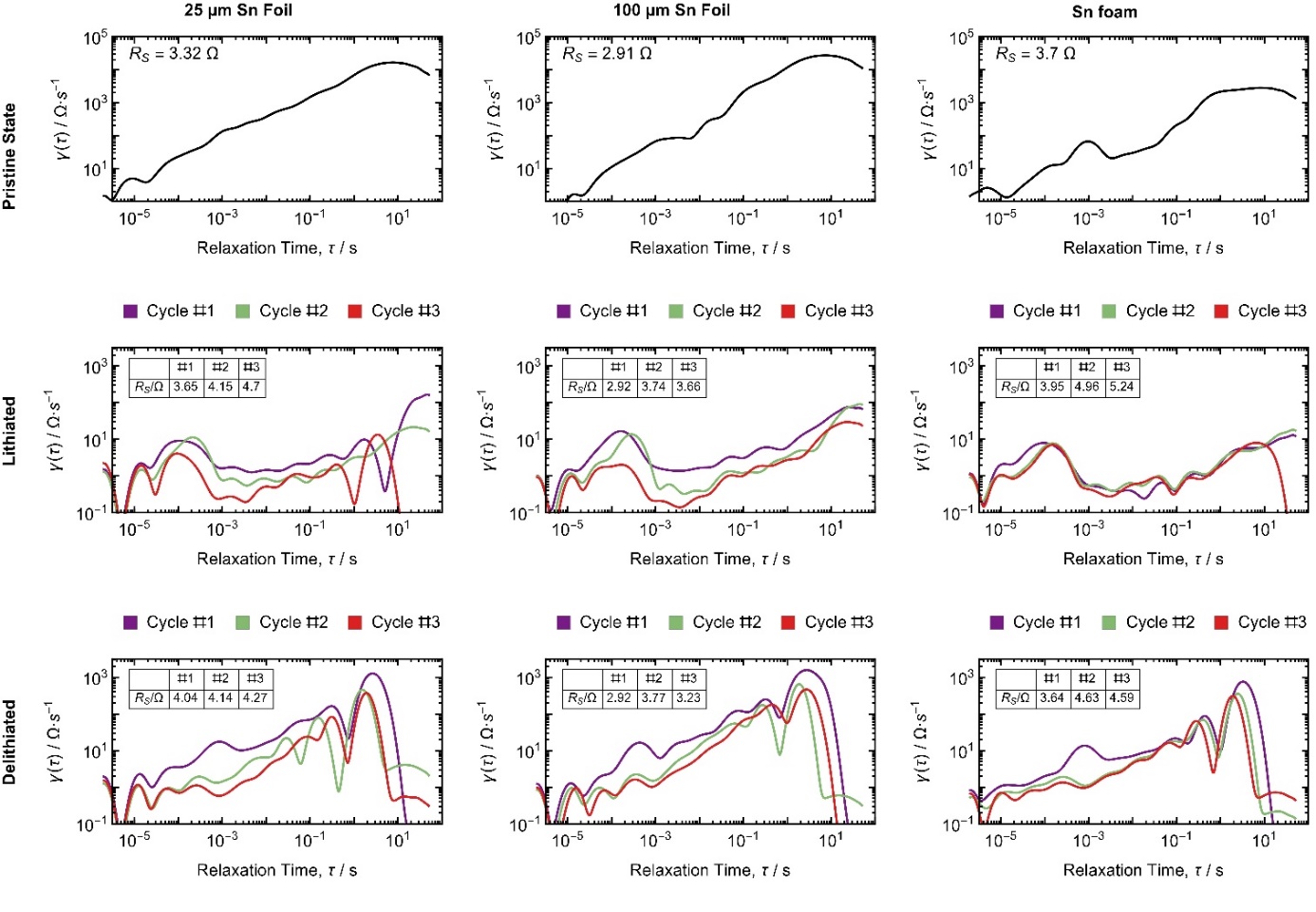


Figure S5: Overview of the distribution of relaxation times function $\gamma(\tau)$ for the EIS data from figures S3 and S4. The Log-Log-plot representation allows for the comparison over a wide amplitude range. The inset tables within the graph display the values of the respective solution resistances $R_{S}$.


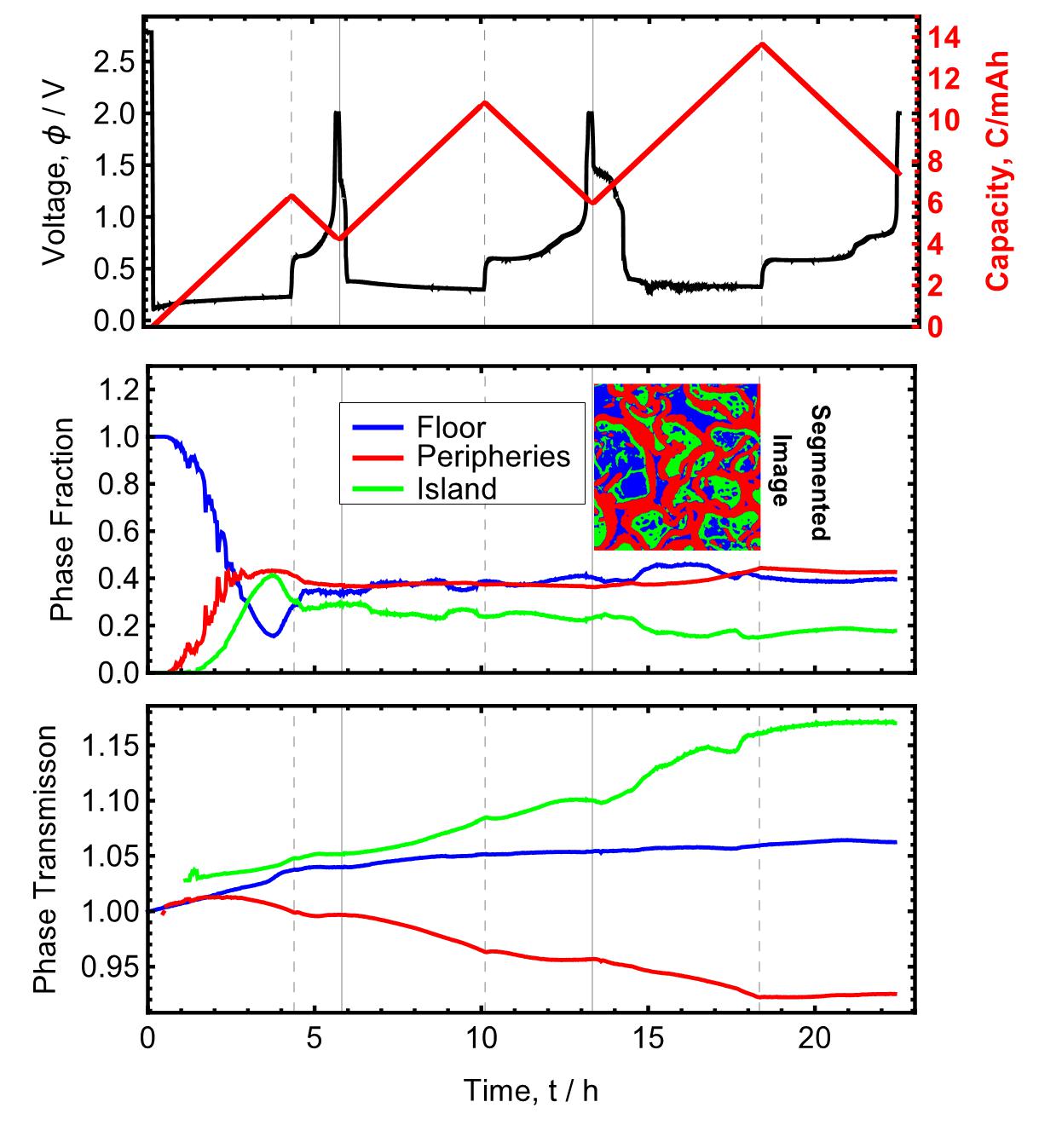


Figure S6: Morphology analysis of the Sn foil electrode (25 µm thickness, 14 mm diameter) cycled in a half-cell configuration against lithium counter-electrode. Top row: voltage profile. Middle row: Phase fraction of the three main morphology features, floor, peripheries, and islands. The inserted image represents a snapshot of the segmentation, Bottom row: Average transmission coefficient of the three regions. The cell was discharged/charged at a current of 1.54 mA, with the lithiation process limited to four hours.


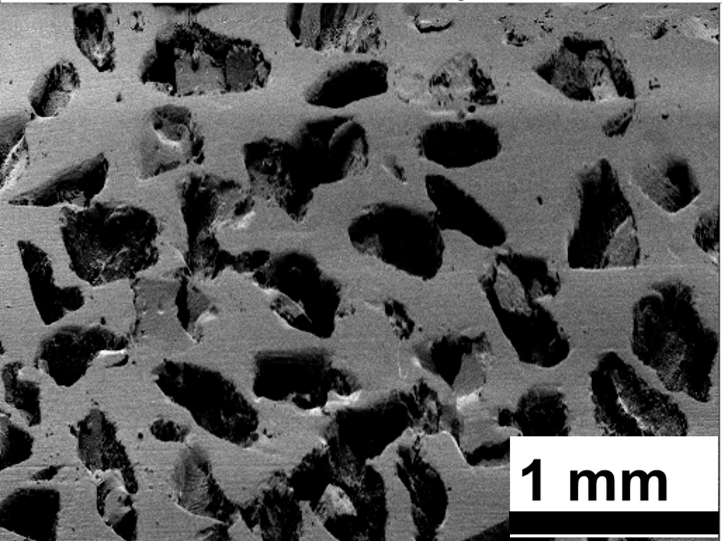


Figure S7: Scanning Electron Microscopy (SEM) image of Sn foam.


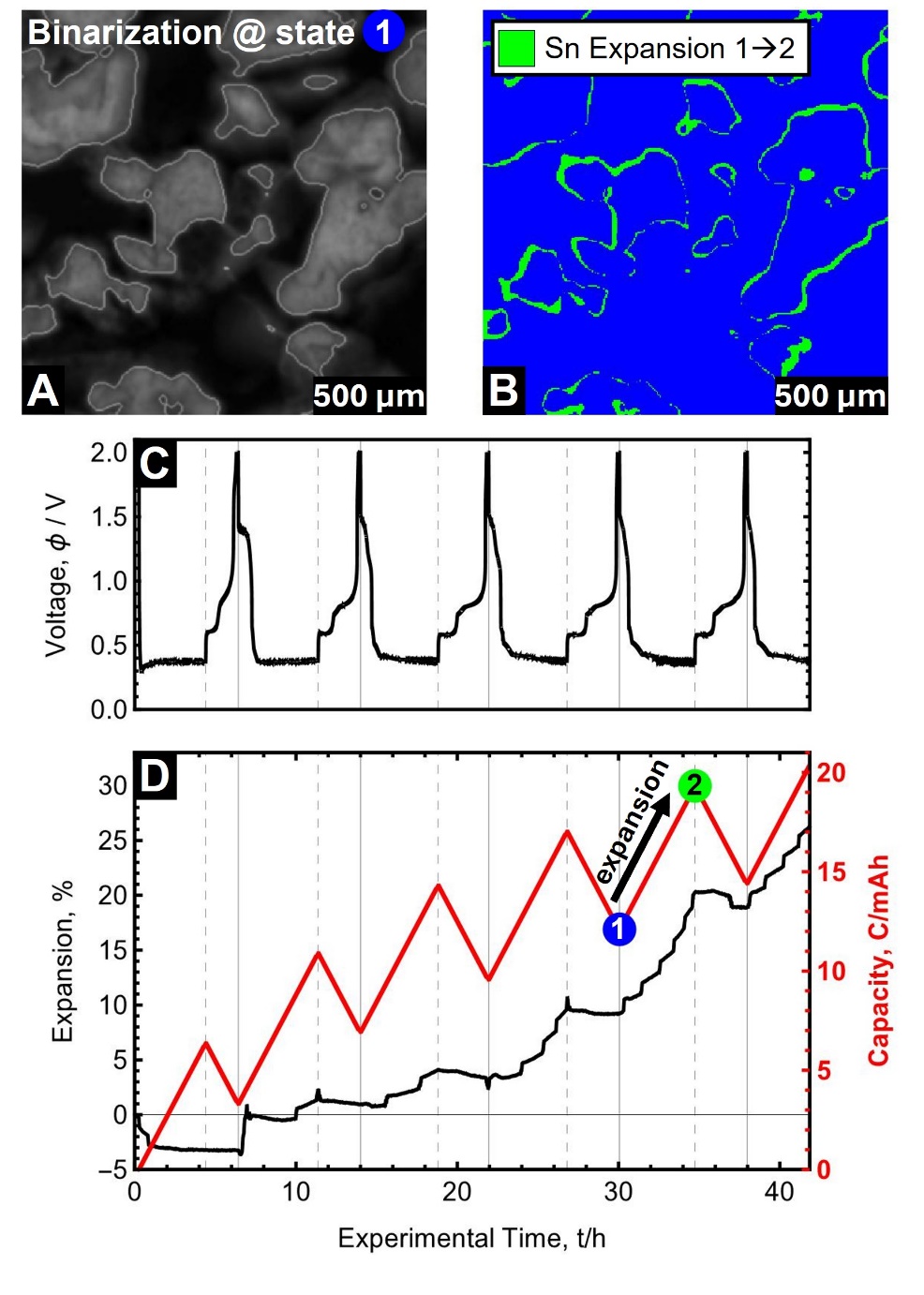


Figure S8: Expansion analysis of the Sn foam. A: Overlay of the binarized image and the original radiography image at state 1 (approx. 30 h of experimental time). B: Visualization of the Sn matrix expansion (green) into the pore volume from state 1 to state 2. C: Voltage profile during the experiment. D: Joint plot of the capacity stored in the Sn foam (red) and the expansion of the Sn bulk (black).

**Expansion Analysis**

The radiography images were binarized to obtain a black (Sn) and white (pore) image (Figure S8A) to measure the relative area fraction of the projected images. The expansion was calculated by normalizing the Sn fraction (black) to the experiment's initial value (0 h). The cell current was integrated to obtain the capacity stored in the bulk Sn phase of the foam (red curve in Figure S8D). For the visualization of the expansion, the difference between the charged state of the 4th cycle and the discharge state of the 5th cycle was calculated, which is shown in Figure S8B.


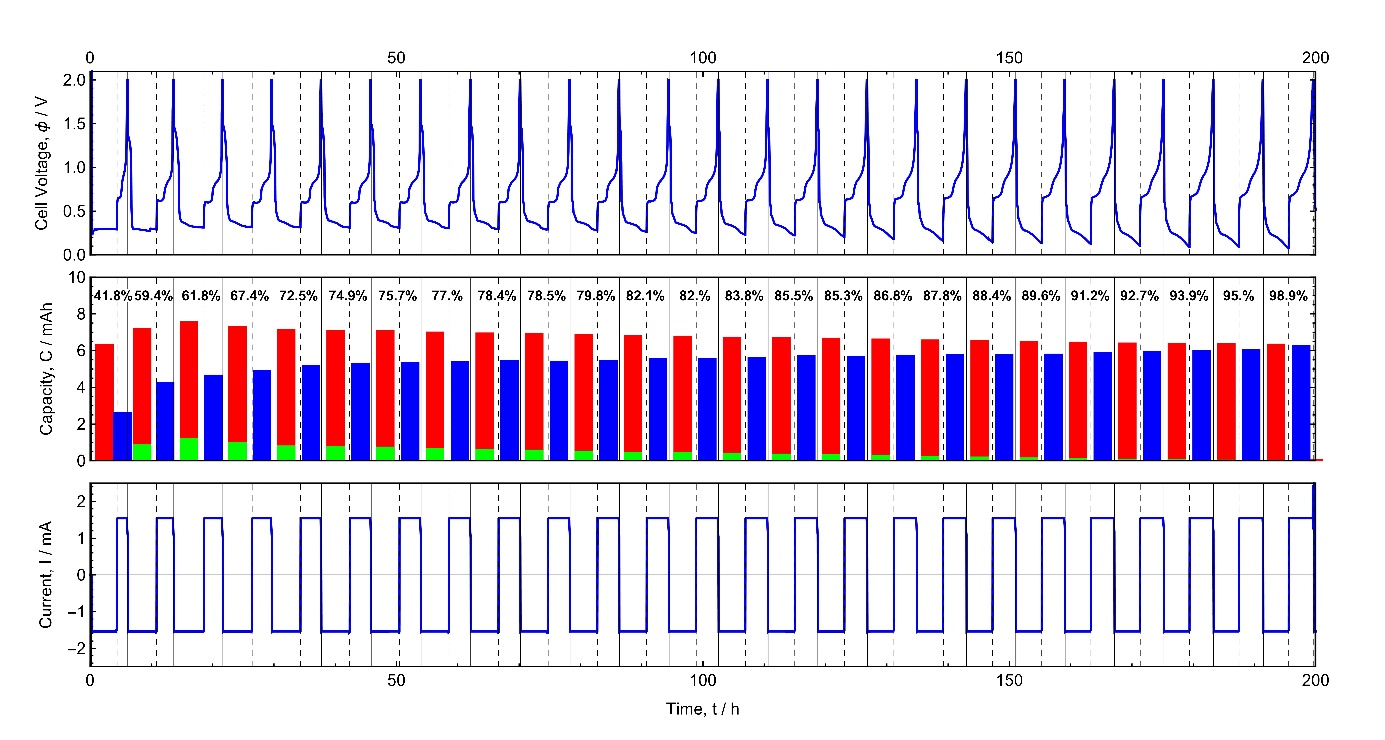


Figure S9: Galvanostatic discharge/charge profiles and the corresponding capacities of the Sn foam electrode (14 mm diameter and 250 µm thickness) cycled in a half-cell configuration against lithium counter-electrode. The cell was discharged/charged at a current of 1.54 mA, with the lithiation process limited to four hours. Capacity: lithiation (green before 0.8 V, red starting from 0.8V), delithiation (blue). The inset values in the capacity plot (middle row) represent the Coulombic efficiencies of their respective cycles.


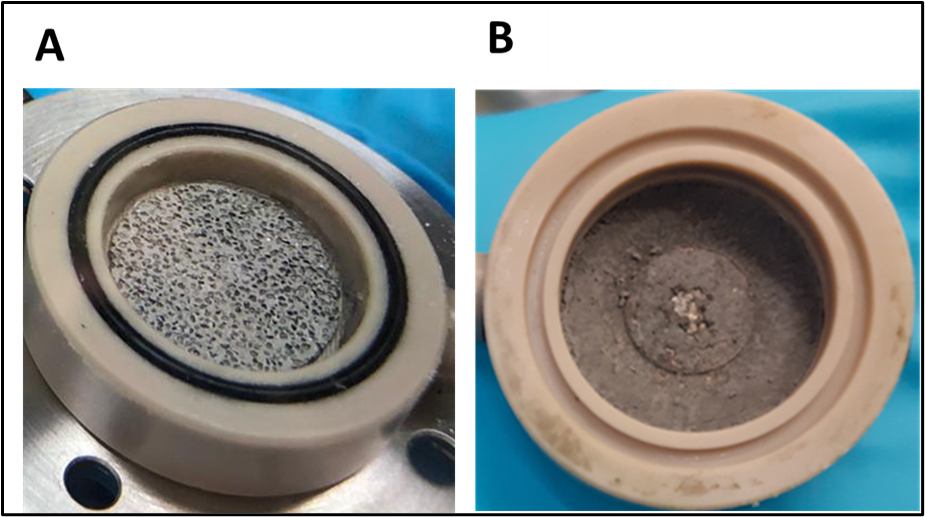


Figure S10: Images of Sn foam electrodes (250 µm thickness and 14 mm diameter): A) pristine and B) after cycling in a half-cell configuration against lithium counter-electrode. For B, the cell was discharged/charged at a current of 1.54 mA, with the lithiation process limited to four hours. This image corresponds to the sample shown in Figure S9 after 25 cycles.


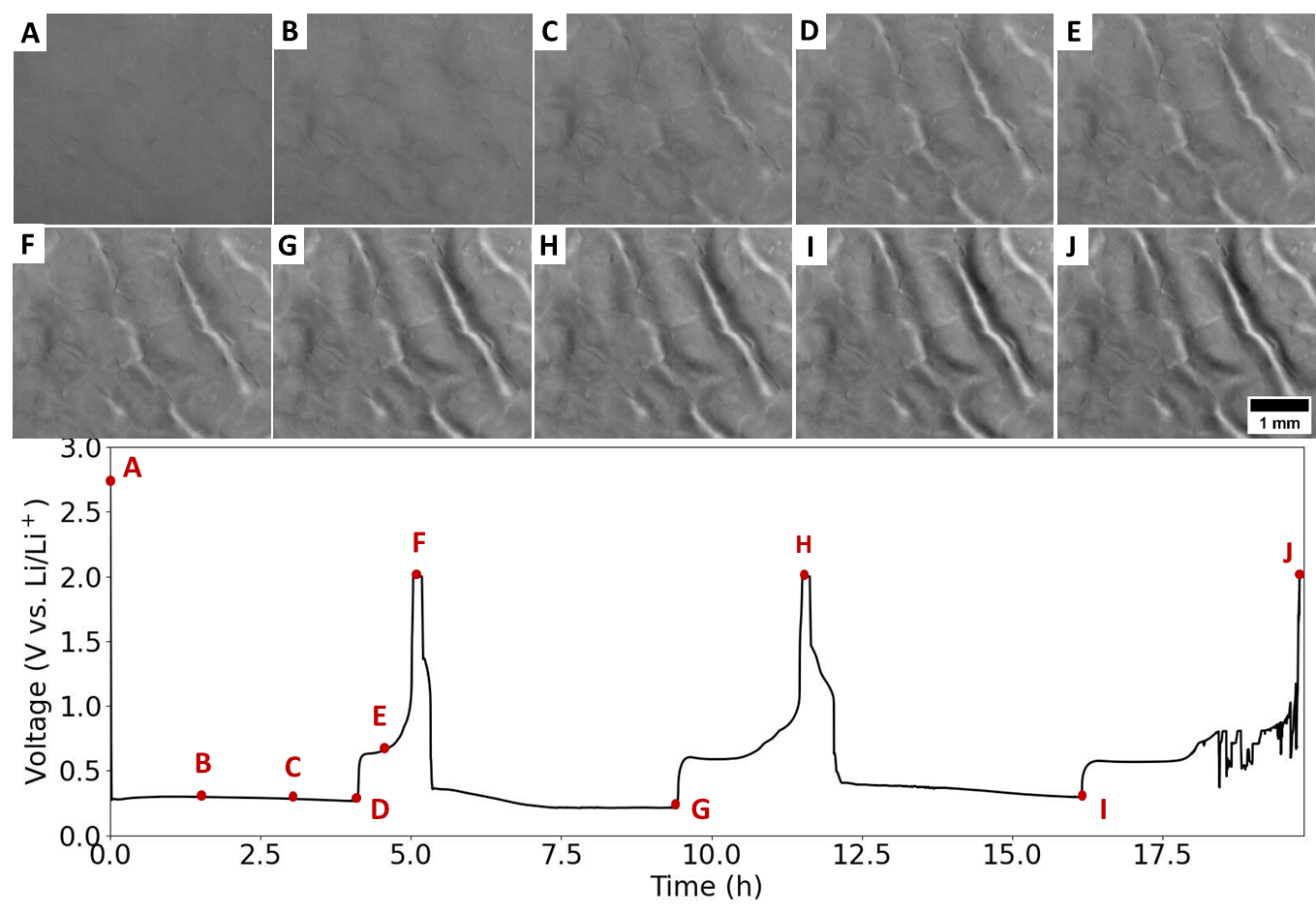


Figure S11: 2D morphological evolution and galvanostatic discharge/charge profiles of the Sn foil electrode (100 µm thick and 14 mm diameter) cycled in a half-cell configuration against lithium counter-electrode during the first three cycles. The cell was discharged/charged at a current of 1.54 mA, with the lithiation process limited to four hours. Frames (A-J): Lab-based X-ray images captured at different stages of lithiation/delithiation process. The specific time points at which these images were acquired are indicated on the corresponding voltage profiles.


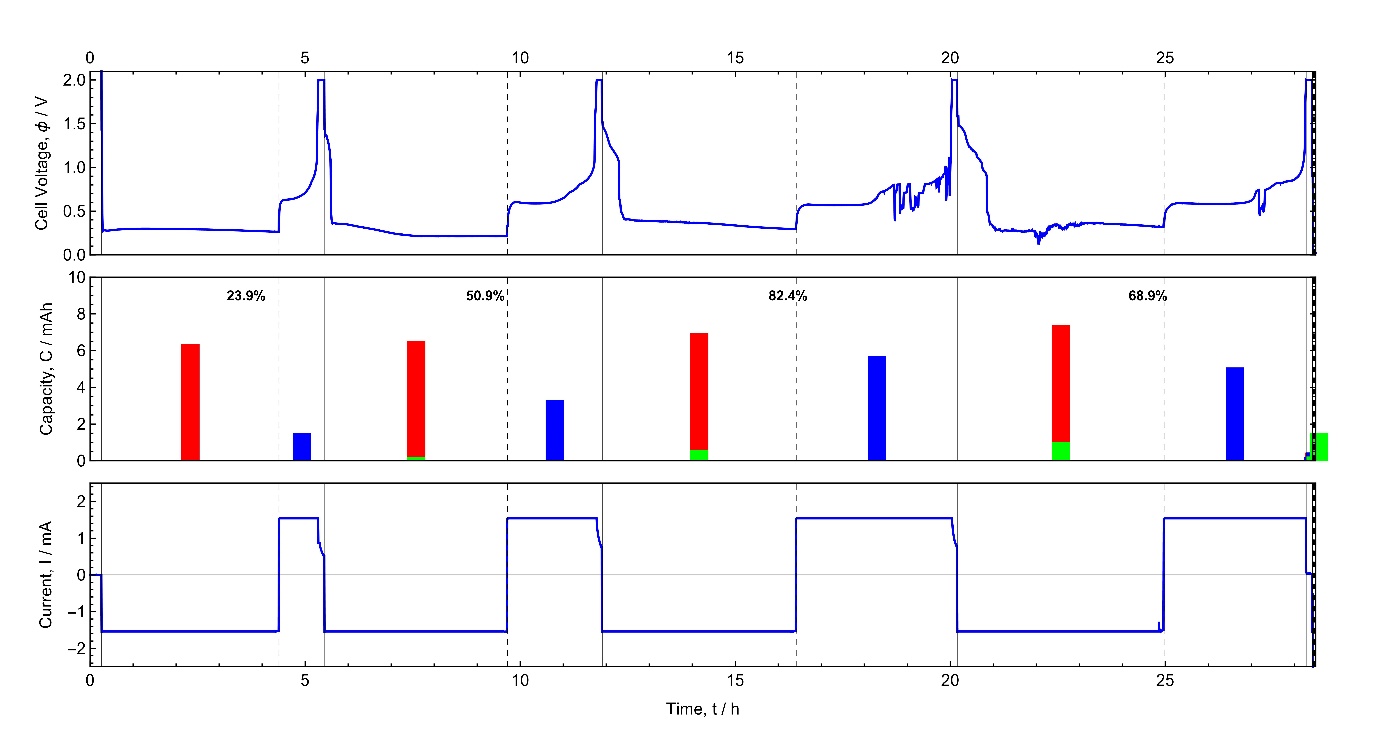


Figure S12: Galvanostatic discharge/charge profiles and the corresponding capacities of Sn foil electrode (100 µm thickness and 14 mm diameter) cycled in a half-cell configuration against lithium counter-electrode. The cell was discharged/charged at a current of 1.54 mA, with the lithiation process limited to four hours. Capacity: lithiation (green before 0.8 V, red starting from 0.8V), delithiation (blue). The inset values in the capacity plot (middle row) represent the Coulombic efficiencies of their respective cycles.
